# 25OHVitamin D Levels in a Canarian Pediatric Population with and without Type 1 Diabetes: The Role of Acidosis

**DOI:** 10.3390/nu15133067

**Published:** 2023-07-07

**Authors:** Yeray Nóvoa-Medina, Marta Barreiro-Bautista, Marta Perdomo-Quinteiro, Jesús María González-Martín, Sofía Quinteiro-González, Ángela Domínguez, María Cabrera, Sara López, Svetlana Pavlovic, Ana M. Wägner

**Affiliations:** 1 Unidad de Endocrinología Pediátrica, Complejo Hospitalario Universitario Insular Materno Infantil, 35016 Las Palmas de Gran Canaria, Spain; sofiaquinteirogonzalez@gmail.com (S.Q.-G.); domgarang@gmail.com (Á.D.); maria.cabrera87@gmail.com (M.C.); sara.lopez0192@gmail.com (S.L.); 2 Asociación Canaria para la Investigación Pediátrica (ACIP Canarias), 35004 Las Palmas de Gran Canaria, Spain; 3 Instituto Universitario de Investigaciones Biomédicas y Sanitarias, Universidad de Las Palmas de Gran Canaria, 35016 Las Palmas de Gran Canaria, Spain; martabarreiro@gmail.com (M.B.-B.); ana.wagner@ulpgc.es (A.M.W.); 4 Facultad de Medicina, Universidad de Las Palmas de Gran Canaria, 35016 Las Palmas de Gran Canaria, Spain; martaperq@gmail.com; 5 Unidad de Investigación, Hospital Universitario de Gran Canaria Dr. Negrín, 35002 Las Palmas de Gran Canaria, Spain; josu.estadistica@gmail.com; 6 Servicio de Pediatría, Complejo Hospitalario Universitario Insular Materno Infantil, 35016 Las Palmas de Gran Canaria, Spain; spavnes@gobiernodecanarias.org; 7 Servicio de Endocrinología y Nutrición, Complejo Hospitalario Universitario Insular Materno Infantil, 35016 Las Palmas de Gran Canaria, Spain

**Keywords:** type 1 diabetes, pediatrics, 25OHVitamin D, acidosis

## Abstract

The role of Vitamin D in the development of type 1 diabetes (T1D) is controversial. The Canary Islands have the highest incidence of childhood-onset T1D in Spain and one of the highest in Europe. We aimed to evaluate 25OHVitamin D concentrations in a Canarian pediatric population, to assess the existence of seasonal variation, to study their association with T1D, and to evaluate the role of acidosis in its levels. In a retrospective, case-control study, we obtained data from 146 T1D patients (<15 years of age) and 346 control children; 25OHVitamin D concentrations were assessed in serum by automatic ChemiLuminescence ImmunoAssay technology. We found significantly higher 25OHVitamin D levels in the summer and autumn months and an inverse correlation between T1D and age; 25OHVitamin D sufficiency was similar in both groups (44.5% vs. 45.1%), with significant differences in the percentage of patients presenting vitamin D deficiency (11.6% (T1D) vs. 16.4% (controls)). When stratified according to the presence of ketoacidosis at sampling, only patients with acidosis showed lower 25OHVitamin D concentrations than controls. Despite its subtropical geographic location, Vitamin D deficiency is frequent in children in Gran Canaria, and 25OHVitamin D concentrations show seasonal variation. After adjusting for acidosis, no differences were found between children with and without T1D.

## 1. Introduction

Type 1 diabetes (T1D) is the most frequent type of diabetes found in children. Its development is related to genetic and environmental factors, including (but not limited to) feeding practices, 25OHVitamin D levels, and infectious diseases [1]. 

Vitamin D is a key hormone in the regulation of calcium and phosphorus metabolism and plays a pivotal role in bone health. It also has effects on extraskeletal tissues, as many cells throughout the body express the vitamin D receptor. In tissues including the brain, heart, pancreas, stomach, gonads, prostate, lymphatics, and skin, vitamin D appears to play a role in improving immune function and reducing inflammation [2], and its deficiency has been associated with the development of atopy and autoimmunity in childhood [3]. It is mainly found in plant (ergocalciferol or vitamin D2) and animal products (cholecalciferol or vitamin D3). Cholecalciferol can also be synthesized in the skin through ultraviolet radiation, hence the importance of the latitude and hours of sunlight exposure in the levels of an individual’s Vitamin D levels. Once ingested, the inactive forms of vitamin D go through hydroxylation in the liver (25 hydroxylation resulting in calcidiol) and kidneys (1 hydroxylation), thus producing the active form of calcitriol [4]. The optimal 25OHVitamin D level and the threshold to define sufficiency are controversial. In 2016, the Global Consensus Recommendations on Prevention and Management of Nutritional Rickets was published as universal guidance to prevent rickets. Based on the Global Consensus, serum 25OHVitamin D level > 20 ng/mL (50 nmol/L) is sufficient to prevent rickets in children and adolescents [5]. However, higher values might be necessary to have an effect on extraskeletal diseases. The prevalence of vitamin D deficiency is considered high throughout the world, even in countries close to the equator, and ranges from 1% to 95% depending on the threshold used to define vitamin D deficiency [6]. New lifestyle habits, current “epidemics” of obesity in children and adolescents worldwide, the use of sunscreen lotions, and other preventable risk factors may play a role in favoring the occurrence of vitamin D deficiency. Studies assessing its relationship with T1D show conflicting results [7]. However, some studies suggest a possible relationship with T1D appearance, progression, and metabolic control [4,8,9]. 

25OHVitamin D serum levels are used to determine vitamin D status due to its long half-life and high concentration, and it is the metabolite used by international societies to establish reference values and recommended dietary reference intake [10]. When evaluating its relationship with T1D, it is important to take into account the moment in which the blood sample was obtained, especially in children with T1D who present with acidosis. It has been described that acidosis (or low levels of bicarbonate) influences the levels of 25OHVitamin D, resulting in lower levels compared to patients with normal pH and bicarbonate levels. The exact mechanisms responsible for this phenomenon have not yet been fully explained [11]. 

Gran Canaria is one of the eight Canary Islands (Spain) located in the Atlantic Ocean, about 100 km off the African coast. With a complex genetic background [12], and an incidence of 30/100,000 children under 14 years of age during the last 15 years, the Canary Islands is the region with the highest reported T1D incidence in Spain and one of the highest in Europe. The current inhabitants result from an admixture of North Africans, western Europeans, and sub-Saharan Africans. European influence comes mostly from the Iberian Peninsula (mostly Galicia and Portugal) and provides close to 70% of the genetic influence. Another 25% comes from North Africa, from the Berber population, and approximately 3% comes from sub-Saharan influence [12]. Being near the Tropic of Cancer, the Canary Islands have great climatic stability throughout the year and are exposed to close to 3000 h of sunlight per year [13], essential for the synthesis of 25OHVitamin D by the skin [14]. Children living in northern latitudes are at risk of vitamin D deficiency, particularly in winter/early spring. Indeed, sufficient summer/autumn 25OHVitamin D status appears important to mitigate such winter nadirs in these children [15]. Given the high incidence of T1D in our region, the possible association between T1D and 25OHVitamin D concentrations, and the lack of data regarding the latter in our pediatric population, we aimed to assess them, as well as the existence of seasonal variation, the association between 25OHVitamin D concentrations and T1D and the role of acidosis in its levels.

## 2. Materials and Methods

### 2.1. Study Design 

We performed a retrospective, case-control study; 25OHVitamin D determination has been part of the initial diagnostic protocol for both T1D and non-diabetic patients in our unit since 2016. In the current paper, we review the data collected since that date for both T1D and a control group. Inclusion criteria:

1. Cases: Patients under 15 years of age diagnosed with T1D in Gran Canaria between 2016 and 2022 and in whom 25OHVitamin D levels were determined at onset (in a minority of patients, 25OHVitamin D levels were not determined). American Diabetes Association (ADA) criteria were used for the diagnosis of T1D [12].
2. Controls: Patients followed in our center’s pediatric endocrinology outpatient clinic between 2016 and 2022, excluding those diagnosed with obesity, T1D, and skeletal and phosphocalcic metabolism disorders.

### 2.2. Data Collection

We collected the following variables: sex, month of diagnosis of T1D for the cases (25OHVitamin D level was determined at that moment), and month in which the first 25OHVitamin D value was obtained for controls. pH and bicarbonate are analyzed in all new onset T1D patients as part of our protocol. Acidosis was defined as a pH below 7.3 and/or bicarbonate levels lower than 15 mmol/L; 25OHVitamin D levels were always obtained after acidosis was corrected in patients with T1D. Age was collected at the moment of blood sampling. These values were obtained throughout the year for both groups, allowing for the evaluation of the existence of a seasonal pattern in our population. Body weight and height were measured, and body mass index (BMI) was calculated within 1 month of onset for patients with T1D and within 3 months of the 25OHVitamin D determination for controls. Seasons were defined as follows: spring: 21 March to 21 June; summer: 22 June to 23 September; autumn: 24 September to 21 December; winter: 22 December to 20 March. 

### 2.3. Laboratory Analyses

25OHVitamin D concentrations (25-hydroxy-vitamin D2 + D3 (DTOT25)) were assessed in serum by automatic ChemiLuminescence ImmunoAssay technology on DiaSorin LIAISON (DiaSorin AB. Stockholm, Sweden); 25OHVitamin D deficiency, insufficiency, and sufficiency were defined as levels < 20, 20–30, and >30 ng/mL, respectively.

### 2.4. Statistical Analyses

We used R Core Team 2022 [13], version 4.2, for statistical analysis. For descriptive statistics, we computed the mean, standard deviation, median, and 25th and 75th percentiles for quantitative variables and the frequency and percentage for qualitative variables. The Kolmogorov–Smirnov test was used to verify the normality of the data based on the sample size. Fisher’s exact test was used to evaluate the dependence of qualitative variables and logistic regression to predict dichotomous variables. Linear mixed-effects models were used to identify factors associated with serum 25OHVitamin D concentrations (the outcome), including seasonality. We added the variable “acidosis” to the linear regression analysis and evaluated the effect of acidosis on 25OHVitamin D levels, given the existence of reports stating a likely effect of acidosis on 25OHVitamin D levels [14]. Significance was established at *p* < 0.05. 

## 3. Results

We obtained data from 146 patients diagnosed with T1D between 2016 and 2022, as well as 346 controls from our outpatient clinic. 25OHVitamin D sufficiency was similar in the T1D and control groups, but patients with T1D were more frequently 25OHVitamin D deficient. BMI was similar in both groups. Table 1 summarizes baseline data. Table 2 summarizes the list of diagnoses from our control group. 

Linear regression analysis showed a significant positive association of 25OHVitamin D levels with season (b = 8.3 for summer and b = 5.1 for autumn, with *p* < 0.001 for both) and an inverse association with age (b = −0.35, *p* = 0.01) and with T1D (b = −2.1, *p* = 0.03) (reflecting increasing levels in the summer and autumn months for both T1D and control groups, and decreasing levels with age and T1D vs. controls) (Table 3). Figure S1 represents the goodness of fit for the model. 

**Figure 1:**
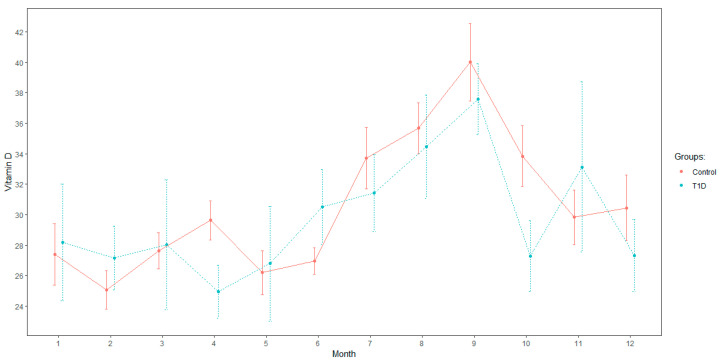
25OHVitamin D levels per month.

**Figure 2:**
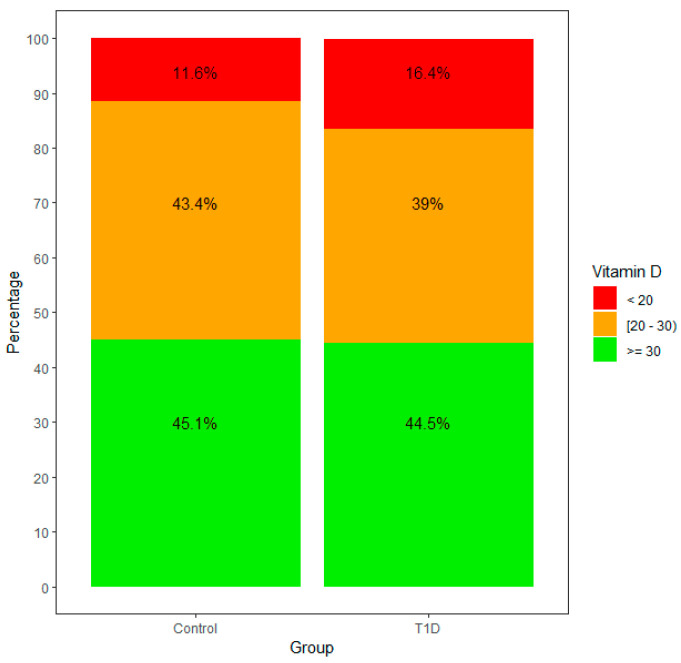
25OHVitamin D levels in patients with T1D and controls.

We then analyzed the same values dividing the outcome to predict the variable 25OHVitamin D as deficient or not deficient (values <2 0 and >20 ng/mL, respectively). With all other variables being similar in both groups (T1D and control), age increases the likelihood of having values under 20 ng/mL (OR = 1.1 per year, *p* = 0.02), children with T1D have an increased risk of having 25OHVitamin D deficiency (OR = 1.9, *p* = 0.02) and summer is the season with the smallest risk of 25OHVitamin D deficiency (OR = 0.24, *p* < 0.001); 25OHVitamin D levels per month are represented in Figure 1. Table S1 summarizes 25OHVitamin D levels per season. Figure 2 represents 25OHVitamin D levels in both groups. 

While still analyzing data from all patients and controls, we added the variable “acidosis” to the linear regression analysis and evaluated the effect of acidosis on 25OHVitamin D levels. We observed lower levels of 25OHVitamin D levels in patients with T1D presenting with ketoacidosis (DKA) at onset compared to those without DKA (26.2 ± 9.1 vs. 31.8 ± 9.2; *p* < 0.0001). We did not observe any differences in 25OHVitamin D levels between patients with T1D who did not present with acidosis and the control group (b = −0.6; *p* = 0.6). Table 4 summarizes the results.

## 4. Discussion

This study evaluated the levels of 25OHVitamin D in a pediatric population with and without T1D in Gran Canaria, as well as the possible existence of seasonal variation. Despite the relatively high sun exposure of the island throughout the year, our results show a clear seasonal pattern, with higher levels in summer and autumn compared to winter months. Regarding the comparison between patients with T1D and healthy controls, after adjusting for possible confounders, in our initial analysis, we observed lower levels in patients with T1D as well as a higher prevalence of 25OHVitamin D deficiency. However, further investigation showed that the difference was only found in those patients who presented with DKA at T1D onset. To our knowledge, this is the first study evaluating 25OHVitamin D levels in children with and without T1D in our region. Furthermore, it is also one of the few to directly evaluate the role of acidosis in 25OHVitamin D levels in children with T1D. 

### 4.1. 25OHVitamin D and T1D

25OHVitamin D’s anti-inflammatory and immunomodulatory effects have led it to be proposed as a preventive measure against T1D [16]. However, its relationship with T1D is still not clear. Studies often look at different effects of Vitamin D on T1D appearance or progression and show mixed results [7]:

- Effect on the appearance of Insulin antibodies: some reports show a reduced risk of developing anti-insulin anti-antibodies in children with higher 25OHVitamin D levels (including TEDDY [17]), whereas other prospective studies such as DAISY and DIADIMMUNE do not confirm the relationship.
- Risk of developing T1D: Interestingly, Finnish studies report reduced relative risk for T1D in children receiving >2000 units of vitamin D/day compared to children receiving <2000 units/day during their first year of life [18]. Furthermore, Stene LC et al. reported a bigger reduction in the risk of developing T1D when supplementation occurs from 7 to 12 months of age compared to supplementation from birth to 6 months of age [8]. Other studies draw attention to the role of polymorphisms in genes involved in vitamin D metabolism. Single nucleotide polymorphisms (SNPs) in *CYP2R1* (25-hydroxylase), *CYP27B1* (1α-hydroxylase), and Vitamin D receptor (*VDR*) genes have been associated with T1D susceptibility [19]. Results from the Spanish population with T1D point in the same direction. In 2005, San Pedro et al. reported a higher frequency of the haplotype “fBAt” in the VDR in Basque patients with T1D compared to healthy controls [20]. Similar results were reported by Martí et al. with patients from Barcelona and Navarra, with certain polymorphisms of the VDR presenting more frequently in patients with T1D compared to controls [21].
- Preservation of residual β cell function: a role for 25OHVitamin D has been suggested in preserving residual β cell function and improving metabolic control in children with recent T1D onset. Panjiyar et al. reported a slower decline in C-peptide and improved metabolic control after 1 year of supplementation with 3000 units of cholecalciferol in children aged 6–12 years in a non-randomized, controlled trial [22]. Gregoriou et al. reported similar results in their systematic review of randomized controlled trials evaluating the effects of Vitamin D supplementation in newly diagnosed T1D patients [4]. They concluded that treatment with alphacalcidol (1-OHVitaminD) and cholecalciferol (but not so with calcitriol) had a positive effect on the daily insulin dose (lower in Vitamin D supplemented T1D patients) and fasting C peptide (higher in Vitamin D supplemented patients). It is important to take into consideration that the follow-up of the studies included in the review ranged from 6 to only 24 months. Additionally, it is important to take into account that other authors have not reproduced these results [19].
- Impact of 25OHVitamin D levels on metabolic control: studies show a correlation between 25OHVitamin D levels, metabolic control, and total insulin dose. Savastio et al. reported a significant correlation between 25OHVitamin D insufficiency and deficiency and HbA1c, with significant improvement in metabolic control after supplementation with 1000 IU/day [9]. However, not all studies report the same results. In a recent systematic review performed by Folino-Nascimento et al., only 50% of the studies included in the review reported significant improvement in glycemic control after vitamin D supplementation [23].

When comparing levels in children with and without T1D, we observed that publications differ widely in their methodology. Some perform cross-sectional studies [24], whereas others don’t take seasonality into account or don’t differentiate between patients presenting with and without acidosis at onset [25,26]. In general, 25OHVitamin D levels are similar between T1D patients and controls, except in those where patients with acidosis at onset are included in the study. 

### 4.2. Prevalence of 25OHVitamin D Deficiency

Latitude and hours of sunlight clearly play a role in 25OHVitamin D levels, with children from higher latitudes presenting lower concentrations and a higher prevalence of vitamin D deficiency [27]. Due to its location, close to the tropic of Cancer (28–29° north of the equator), the Canary Islands present a mild year-round temperature (average temperature 21 °C (17.9–24.6°)), with many hours of sunlight throughout the year, ranging from 184 in January to 308 in July [28]. Although finding such a high prevalence of 25OHVitamin D insufficiency and deficiency (55%) might be unexpected, other studies with children living in similar latitudes have reported equivalent results. Studies from Qatar, Indonesia, and Israel also show a rather high prevalence of 25OHVitamin D insufficiency (40 to 80%) [29,30,31], as well as evidence for seasonality [31]. Bener et al. reported a prevalence of 25OHVitamin D deficiency of 61% in Qatari adolescents, 28.9% in 5–10-year-olds, and 9.5% in younger children [29]. Pulungan et al. reported a prevalence of 25OHVitamin D insufficiency of 37.5% in children aged 7 to 12 years from Indonesia and only 1.7% in 25OHVitamin D deficiency [30]. Brody et al. reported a prevalence of 25OHVitamin D insufficiency of 62.3% (defined in their study as 15–29 ng/mL) and deficiency of 18.1% (defined as levels of 25OHVitamin D <15 ng/mL) in Israeli children and adolescents with T1D [31]. These reports highlight the elevated number of children presenting suboptimal levels of 25OHVitamin D worldwide. Recent data from the north of Spain show a prevalence of 25OHVitamin D insufficiency and deficiency (<30 ng/mL) of 56.3%, with only 43.7% of children presenting normal levels (similar to our results) [32]. They also showed that in the study population, diet has a limited role in the presence of 25OHVitamin D insufficiency, with factors such as sun exposure or ethnicity playing a greater role. The use of sunscreen during the summer months, less hours spent outdoors during winter and spring (in spite of the relatively mild winters) [26], the high prevalence of obesity [33] in our population [34], along with the lack of vitamin D food fortification [35] probably account for the low levels reported in the present study. 

Given the elevated prevalence of 25OHVitamin D deficiency and insufficiency (both in children with and without T1D), and the role supplementation might play in preserving β cell function and improving metabolic control, we think that it is important to evaluate 25OHVitamin D in children with T1D; 25OHVitamin D deficiency/insufficiency correction has proven safe [10], and given its potential effect in optimizing metabolic control, supplementation should be considered.

### 4.3. Effect of Acidosis on 25OHVitamin D

Those studies that evaluate the effect of acidosis on 25OHVitamin D levels, in general, report lower levels in patients presenting with acidosis. Choe et al. recently reported lower levels of 25OHVitamin D in children with T1D compared to controls (16 vs. 19.9 ng/mL; *p* < 0.001) [11]. In agreement with our results, they did not find any differences between the T1D group presenting without acidosis and the control group (17.6 vs. 19.9 ng/mL), whereas the difference remained in the T1D group with acidosis compared with controls (15 vs. 19.9 ng/mL; *p* < 0.001).

Al-Zubeidi et al. also reported similar results to ours [36]. In their cohort (185 patients with T1D from San Diego and no control group), they reported 25OHVitamin D sufficiency in only 42% of their patients with T1D (close to our 44.5%) and significantly lower 25OHVitamin D concentrations in patients with acidosis, with increasing age and with sampling in the winter months. Furthermore, they compared 25OHVitamin D levels in some of their patients with acidosis at the moment of diagnosis and 2–3 days later. They observed a mean difference of 5 ng/mL (*p* = 0.04), implying that the presence of low levels of HCO_3_ has an effect on the measurement of 25OHVitamin D levels. The positive correlation between HCO_3_ levels at onset and 25OHVitamin D has also been reported by other authors [37,38]. 

A clear explanation of the pathophysiological pathways leading to the reported difference in 25OHVitamin D levels in patients with DKA at the moment of T1D onset is still lacking. Choe et al. explored in some depth the levels of total, free, and bioavailable 25OHVitamin D, as well as vitamin D binding protein (VDBP) [11]. They concluded that T1D patients presented significantly lower total but higher free 25OHVitamin D levels than healthy children. Additionally, patients presenting with DKA showed lower levels than children without DKA, with a significant association with DKA severity at diagnosis. In relation to VDBP, T1D patients had significantly lower levels than controls, probably related to their higher free 25OHVitamin D levels. They discussed decreased production or increased excretion as possible mechanisms behind the decreased protein levels. These studies emphasize the importance of taking into account HCO_3_ levels (or at least the presence of acidosis) in the evaluation of 25OHVitamin D levels in children with T1D. 

We acknowledge that our study presents some limitations. Its retrospective nature limits the conclusions that can be drawn. Additionally, we did not study other Vitamin D metabolites or VDBP, thus limiting our ability to analyze possible factors resulting in the lower levels detected in children with DKA at the moment of T1D onset. 

To summarize our findings, 25OHVitamin D deficiency and insufficiency are frequent in the Canary Islands in children with and without T1D, in line with findings reported by other authors. Levels present clear seasonal variation, with lower levels in the winter and spring months. We also found lower levels in older children and, initially, in patients with T1D compared to controls. However, the difference is due to lower 25OHVitamin D levels in T1D patients presenting with acidosis, probably related to biochemical mechanisms linked to the effect low HCO_3_ levels have on 25OHVitamin D kinetics or the measurement of its concentrations and not to a real difference in 25OHVitamin D levels between patients with T1D and controls. This final consideration is important when evaluating and reporting 25OHVitamin D levels in patients with T1D, helping to avoid misinterpretation of the results and erroneous conclusions. 

## Figures and Tables

**Table 1:** Baseline data from T1D and control groups.

	T1D	Control	*p*-Value
N	146	346	
Age (years)	8.4 (3.9)	10 (2.7)	<0.001
Sex (% female)	43	57	0.006
25OHVitamin D (ng/mL)	29.4 (10.4)	30.8 (10.4)	0.17
25OHVitamin D (%>30/<20ng/mL)	44.5/11.6	45.1/16.4	
BMI (Kg/m^2^) (median(IQR))	17.3 (4.5)	17.6(6.1)	0.8 *

T1D: Type 1 diabetes; BMI: body mass index. IQR: inter quartile range * Mann Whitney U test. If not stated otherwise, variables are described as mean (sd).

**Table 2:** Summary of diagnoses for control patients.

Diagnosis	N
Short stature	103
Precocious Puberty and other pubertal disorders	94
Congenital Adrenal Hyperplasia	25
Thyroid disorders	28
Isolated non-diabetic hyperglycemia	18
Hypoglycemia	3
Gender dysphoria	4
Hypercholesterolemia	5
No endocrine disorder	16
Others	21

**Table 3:** Linear regression analysis.

Variables	Multivariate Analysis			
	b	B	CI (95%)	pval
Intercept	31.37	-	27.62–35.13	<000.1
Age	−0.35	−0.11	−0.64–−0.07	0.01
Sex: F	−1.34	−0.06	−3.13–0.46	0.14
Group: T1D	−2.18	−0.1	−4.17–−0.2	0.03
Season: Winter	(ref)			
Season: Spring	0.92	0.04	−1.55–3.4	0.46
Season: Summer	8.34	0.37	5.9–10.77	<000.1
Season: Autumn	5.11	0.2	2.46–7.76	<000.1
AIC	3643.33			
Adjusted R^2^	0.12			

F: female; T1D: Type 1 diabetes; b: unstandardized regression coefficient; B: standardized coefficient; CI(95%): 95% confidence interval; pval: *p* value. AIC: Akaike information criterion.

**Table 4:** Linear regression analysis, including acidosis.

Variables	Multivariate Analisis (All Variables)				
	b	EE	B	IC (95%)	pval
Intercept	31.57	1.91	-	27.8–35.3	<000.1
Age	−0.38	0.15	−0.12	−0.6–−0.09	0.009
Sex: F	−1.14	0.91	−0.06	−2.9–0.6	0.207
Control group	(ref)				
T1D with acidosis (N = 59)	−5.31	1.41	−0.17	−8–−2.5	<000.1
T1D without acidosis (N = 79)	−0.63	1.23	−0.02	−3–1.78	0.608
Season: Winter	(ref)				
Season: Spring	0.77	1.25	0.03	−1.6–3.2	0.54
Season: Summer	8.4	1.22	0.37	6–10.8	<000.1
Season: Autumn	4.95	1.34	0.19	2.3–7.5	<000.1
AIC	3567.15				
Adjusted R^2^	0.14				

F: female; T1D: Type 1 diabetes; b: unstandardized regression coefficient; B: standardized coefficient; CI(95%): 95% confidence interval; pval: *p* value. AIC: Akaike information criterion.

## Data Availability

Data is unavailable due to privacy or ethical restrictions.

## Supplementary Materials

The following supporting information can be downloaded at: https://www.mdpi.com/article/10.3390/nu15133067/s1, Figure S1: Goodness of fit; Table S1: 25OHVitamin D levels per season in both groups.
