# Supplementary material for: 25OHVitamin D Levels in a Canarian Pediatric Population with and without Type 1 Diabetes: The Role of Acidosis
Source: Nutrients. 2023 Jul 7;15(13):3067. doi: 10.3390/nu15133067 (PMC10346717; doi:10.3390/nu15133067)
Supplement: Supplementary file 1 [file nutrients-15-03067-s001.zip › nutrients-2439008-supplementary.pdf]

Supplementary materials

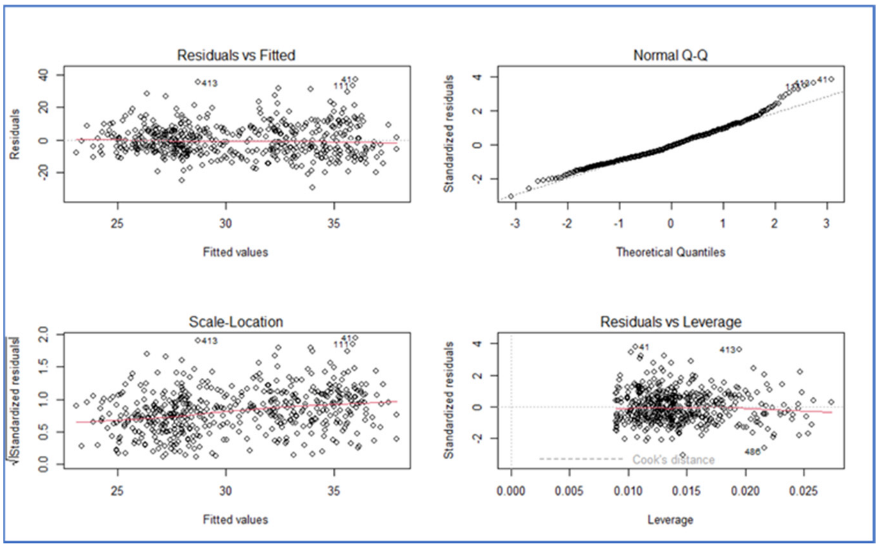

Figure S1. Goodness of fit.

25OHVitamin D	Control-Winter	T1D-Winter	Control-Spring	T1D-Spring	Control-Summer	T1D-Summer	Control-Autumn	T1D-Autumn
N	77	33	95	39	104	42	70	32
Mean	27	25.78	27.39	27.95	35.5	33.35	32.72	29.75
Standard Deviation	7.83	8.87	6.54	10.79	12.45	10.85	10.67	9.46
Min	7.4	9.2	15.1	3	12.8	4.4	13.6	13.2
P25	20.6	21.4	22.35	20.85	26.08	26.13	24.42	19.88
Median	26.6	24.2	26.1	27.5	32.6	32.3	32.55	31.2
P75	31.4	31.5	31.6	32.2	45.12	40.53	38.68	36.57
Max	54.2	44.8	49.6	64	73.1	57.2	63.9	49

Table S1. 25OHVitamin D levels per season in both groups.
